# Novel PCR-Based Multiplex Assays for Detecting Major Quality and Biotic Stress in Commercial and Weedy Rice

**DOI:** 10.3390/life12101542

**Published:** 2022-10-04

**Authors:** Noraikim Mohd Hanafiah, Acga Cheng, Phaik-Eem Lim, Gomathy Sethuraman, Nurul Amalina Mohd Zain, Niranjan Baisakh, Muhamad Shakirin Mispan

**Affiliations:** 1 Institute of Biological Sciences, Faculty of Science, Universiti Malaya, Kuala Lumpur 50603, Malaysia; 2 Institute of Ocean and Earth Science, Universiti Malaya, Kuala Lumpur 50603, Malaysia; 3 School of Plant, Environmental and Soil Science, Louisiana State University Agricultural Center, Baton Rouge, LA 70803, USA

**Keywords:** agarose gel electrophoresis, amylose content, bacterial leaf blight, blast, fragrance, multiplex polymerase chain reaction, rice, sheath blight, weedy rice

## Abstract

**Simple Summary:**

Rice, the staple food for more than half of humanity, is grown predominantly in Asia, the world’s most populous continent with the fastest-growing economy. The present-day rice industry must not only meet increasing demand but also changing consumer demands, with a strong emphasis placed on producing high-quality rice. While the rapid development of advanced genotyping methods can be useful for modern rice breeding programs, some methods (such as capillary electrophoresis or sequencing) can be costly to apply in laboratories with limited resources. To address this issue, we developed six novel multiplex polymerase chain reaction (PCR) assays that employ a standard agarose-based gel electrophoresis system to simultaneously detect at least two major grain quality (amylose content and fragrance) and biotic stress (blast, sheath blight, and bacterial leaf blight) genes in rice. One of these assays, which can detect all three targeted biotic stresses, was found to be useful in screening Malaysian weedy rice that may contain novel sources of disease resistance. The universal protocol described in this study can be used in routine molecular laboratories to aid rice breeding initiatives in Malaysia and other resource-constrained countries.

**Abstract:**

While previous research has demonstrated that multiplex polymerase chain reaction (PCR) can be a cost-effective approach to detect various genes in crops, the availability of multiplex assays to simultaneously screen both grain quality and biotic stress resistance traits in rice (*Oryza sativa*) is limited. In this work, we report six novel multiplex assays that use a universal protocol to detect major rice grain quality (amylose content and fragrance) and biotic stress (blast, sheath blight, and bacterial leaf blight) traits with amplified products consisting of up to four primer pairs that can be analyzed using a standard agarose-based gel electrophoresis system. Recent studies have suggested that weedy rice has novel sources of disease resistance. However, an intensive screening of weedy biotypes has not been reported in Malaysia. Accordingly, we employed one of the developed multiplex assays to screen reported genes or quantitative trait loci (QTLs) associated with blast, sheath blight, and bacterial leaf blight diseases in 100 weedy rice biotypes collected from five local fields, with phenotyping performed to validate the genotyping results. In conclusion, our universal multiplex protocol is effective for the large-scale genotyping of rice genetic resources, and it can be employed in routine molecular laboratories with limited resources.

## 1. Introduction

Most rice (*Oryza sativa* L.) is grown in Asia, the world’s most populous continent with the fastest-growing economy [1]. The present-day rice industry must not only meet increasing demand but also changing consumer demands, with a strong emphasis placed on producing high-quality rice, which significantly impacts both palatability and consumer acceptance. Fragrance (or aroma) is one of the most important grain qualities of rice [2,3]. According to Hu et al. [2], consumers prefer pleasantly fragranced rice, such as Basmati rice, which has had a large market in Asia for decades despite being more expensive than nonaromatic rice. In addition, a major grain quality feature that has been consistently improved to satisfy changing customer demand is the amylose content, which strongly influences the cooking and eating quality of rice [4,5].

Some developing Asian countries are still reliant on imported rice to meet their needs because they are yet to achieve 100% rice self-sufficiency. For example, Malaysia, which imports rice principally from Pakistan, Thailand, and Vietnam, has only achieved approximately 65% self-sufficiency for its total 2.7-million-ton rice demand as of the early 2020s [6,7]. Rice production in Malaysia has been negatively impacted by several biotic stresses, notably blast, sheath blight, and bacterial leaf blight diseases caused by the pathogens *Magnaporthe oryzae, Rhizoctonia solani,* and *Xanthomonas oryzae*, respectively [8,9]. Climate change is expected to worsen these issues by generally increasing the risk of pathogens spreading in rice fields and agricultural ecosystems, thus potentially leading to greater yield losses [10]. As a result, it is essential to devise radical yet dependable strategies to boost rice production, quality, and resilience [11].

In addition to climate-induced stresses, the presence of weedy (or obnoxious red) rice has been reported to negatively impact rice production in several major rice-growing regions worldwide, including Malaysia [12,13]. Recent research, however, has revealed that weedy rice contains novel sources of stress tolerance or resistance, including to some of the most devastating rice diseases, such as blast, which can eradicate up to 30% of the crop annually [14]. While weedy rice may outcompete cultivated rice for resources, its competitive ability and adaptive evolutionary traits, such as stress tolerance and increased seed dispersal, may be useful to maximize cultivated rice resource use efficiency and yields in the face of climate change [15]. One viable approach to incorporate resilience into modern rice with narrow genetic backgrounds is the introgression of alleles from weedy rice with novel genes that respond to climatic stresses [16]. Although the merits of weedy rice have been deliberated, challenges remain for the utilization of weedy genetic resources due to the paucity of research in this field and the current lack of understanding concerning the biotype variability at different locations within a country or region. Nonetheless, the availability of the complete rice genome sequence and advances in genetics may help to close this gap [11,16].

**Figure 1:**
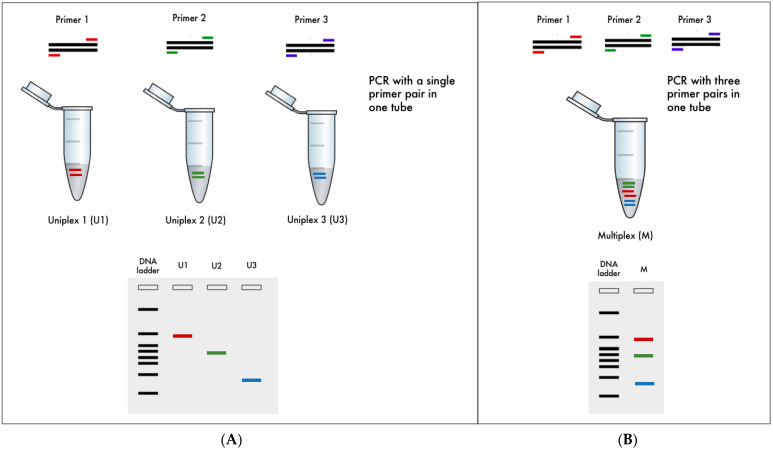
Overview of gel-electrophoresis-based (**A**) uniplex PCR and (**B**) multiplex PCR using three primer pairs.

Multiplex polymerase chain reaction (PCR) is a PCR variant that employs two or more pairs of molecular markers or primers to simultaneously screen multiple genes or loci in a single PCR system. It is a notable example of a common molecular technique that has been effectively applied to crop improvement in recent decades [17,18,19]. Figure 1 illustrates gel-electrophoresis-based uniplex PCR and multiplex PCR with three different primer pairs. Despite prior research demonstrating that multiplex PCR can be a cost-effective and efficient assay to detect various genes in crops, the availability of assays to simultaneously screen major rice grain quality and biotic stress resistance traits is limited or nonexistent; in addition, to our knowledge, there are no reports of their utilization or efficacy in detecting multiple major disease resistance genes in weedy rice [19]. Thus, this is where the novelty of this study resides. The present study aimed to develop multiplex PCR assays that can simultaneously detect at least two major grain quality (amylose content and fragrance) and biotic stress (blast, sheath blight, and bacterial leaf blight) genes in rice using standard agarose gel electrophoresis. One of the six developed multiplex assays, which can detect all three biotic stresses, was found to be useful in screening Malaysian weedy rice (*O. sativa* f. *spontanea*). All assays developed in this study can be employed in routine molecular laboratories, thereby assisting rice breeding initiatives in Malaysia and other developing countries with limited resources.

## 2. Materials and Methods

### 2.1. Materials

The present study included ten local cultivated rice varieties with diverse grain quality and disease resistance backgrounds as well as one hundred weedy rice biotypes collected in three Malaysian states, including Pahang, Selangor, and Perak. The seeds for the cultivated varieties were obtained from the Malaysian Agricultural Research and Development Institute (MARDI). MARDI also provided the isolates of *X. oryzae* (Xoo), *M. oryzae*, and *R. solani* used in this study for weedy phenotyping.

### 2.2. Molecular Analyses

#### 2.2.1. DNA Isolation

For each genotype, approximately 5 g of seeds were sown and grown in a greenhouse at Rimba Ilmu, Universiti Malaya (3.1311° N, 101.6578° E), and leaves of 4-week-old seedlings were harvested for DNA isolation. Total genomic DNA was extracted using a FavorPrepTM Plant Genomic DNA Extraction Mini Kit following the manufacturer’s protocol (Favorgen, Ping Tung, Taiwan). DNA quality was assessed using 1% SYBR® Safe (ThermoFisher Scientific, Waltham, Massachusetts, USA) DNA-stained agarose gel electrophoresed in 1xTris-acetate-ethylenediaminetetraacetic acid (TAE) buffer for 60 min and was visualized under the AlphaImager Mini Imaging System (ProteinSimple, Santa Clara, California, USA). The purity of the samples was also determined using a NanoDrop 2000 spectrophotometer (Thermofisher Scientific, Waltham, Massachusetts, USA). To prepare both uniplex and multiplex PCR samples, 2XGoTaq Green PCR Mastermix (Promega, Madison, WI, USA) was used, which contains optimal concentrations of bacterial-derived *Taq* DNA polymerase, MgCl_2_, dNTP, and reaction buffers.

#### 2.2.2. Selection of Molecular Markers

Table 1 presents the details of five molecular markers selected in this study, which are associated with rice amylose content, fragrance, bacterial leaf blight, blast, and sheath blight resistance [19,20]. The markers were selected based on the availability of genotyping data in the Gramene database (https://www.gramene.org (accessed on 11 September 2019), their degree of polymorphisms, and their melting temperature (55 °C). Although the efficiency of each selected marker has been reported in previous studies (Table 1), they have not been multiplexed in the way that was performed in this study.

#### 2.2.3. Uniplex and Multiplex Polymerase Chain Reaction (PCR)

Each of the five selected primer sets were analyzed individually via uniplex PCR using the Veriti 96-well thermal cycler (Applied Biosystems, Waltham, MA, USA) (Table 2). To validate the results obtained from the uniplex PCR analyses, the amplified products were sequenced using an ABI3100 DNA sequencer (Applied Biosystems, Waltham, MA, USA) in a commercial sequencing facility (Apical Scientific, Seri Kembangan, Malaysia).

Multiplex PCRs were performed on commercial rice varieties using at least two primer pairs associated with two or more quality and disease resistance traits (Supplementary Material Table S1), with distinct banding patterns between positive and negative controls. To screen weedy rice biotypes, multiplex PCR assays with at least two primer pairs linked to disease resistance traits that displayed distinct banding patterns between positive and negative controls were utilized. Each PCR sample contained a total reaction volume of 20 µL, which included 10 µL of premixed, ready-to-use 2XGoTaq Green PCR Mastermix (Promega, Madison, WI, USA), 0.4 mL of each primer, 50 ng of template DNA, and double-distilled water. Table 3 shows the control varieties and the expected product sizes for each of the target-trait primer pairs.

### 2.3. Gel-Based Genotyping

Uniplex and multiplex PCR products were electrophoresed on SYBR^®^ Safe-stained (Thermofisher Scientific, Waltham, MA, USA) 3% standard agarose and 4% high-resolution agarose, respectively, and visualized under the AlphaImager Mini Imaging System (ProteinSimple, Santa Clara, CA, USA). The gels were prepared by mixing 4.5 g of standard agarose (Hydragene, USA) and 6 g of high-resolution agarose (Gene Xpress, Subang Jaya, Malaysia) in 150 ml of 1 × TAE buffer, and amplified products were electrophoresed for 2.5 h at 100 V (for standard agarose gel) or 3 h at 120 V (for high-resolution agarose gel). The sizes of the products was estimated using 100 bp and 50 bp DNA size markers (SMOBIO, Hsinchu, Taiwan).

### 2.4. Phenotyping for Biotic Stresses in Weedy Rice Biotypes

Phenotyping for cultivated rice varieties was not conducted because the phenotypes of the control and some selected varieties for the targeted traits had been reported in earlier studies [19,20,26,27]. Therefore, only the weedy rice evaluated in this study was phenotyped. As a control, two rice checks (MR219 and Ria) were included. The severity of each targeted disease was determined based on the Standard Evaluation System scale (SES) [28] formulated by the International Rice Research Institute (IRRI) (Table 4).

#### 2.4.1. Blast

An inoculum of *M. oryzae* was prepared following Hayashi et al. [29]. Seedlings at the 5-6 leaf stage were inoculated by spraying with a conidial suspension containing 10^5^ (100,000) conidia per ml, as determined by a hemacytometer. To aid the adhesion of the inoculum to the leaves, a drop of Tween 20 was added. Reactions to blast disease were graded following the SES scale (Table 4).

#### 2.4.2. Sheath Blight

An inoculum of *R. solani* was prepared following Jia et al. [30]. To ensure that the presence of the soilborne *R. solani* inoculum had no effect on our results, the soil was first sterilized with steam. Each seedling was inoculated with a mycelial disc, which was placed at the base of the stem and forced up to allow the mycelium to touch the plant. Sheath blight disease reactions were classified according to the SES scale (Table 4) as well as by measuring lesion length.

#### 2.4.3. Bacterial Leaf Blight

An inoculum of *X. oryzae* (Xoo) was prepared following Ke et al [31]. The leaf clipping method described by Kauffman et al. [32] was used to inoculate the bacteria along 4–5 cm of the leaf tip. The inoculation was performed in the afternoon, between 03:00 p.m. and 05:00 p.m., to avoid high environmental heat and evaporation. Reactions to bacterial leaf blight disease were scored based on the SES scale (Table 4) and lesion length measurements [33]. 

### 2.5. Data Analysis

Genotype data were used to calculate parameters related to genetic diversity, including the observed allele number per sampling location (Na), the number of effective alleles (Ne), the observed heterozygosity (Ho), the expected heterozygosity (He), the unbiased expected heterozygosity (uHe), and Shannon’s diversity index (I), using GenAlEx 6 [34,35]. 

## 3. Results

### 3.1. Uniplex PCR and DNA Sequencing

The current study tested five sets of molecular markers that had previously shown functional polymorphisms for each of the target traits, including *Wx*-SSR, *fgr*-SNP, pTA248, RM8225, and RM202 [19,20,21,22,23,24]. All of the selected markers showed functional polymorphisms with expected sizes of amplified products (Table 5), indicating that they can be employed for multiplexing. The amplified regions on local checks (Table 3) were analyzed using both uniplex PCR and sequencing (Supplementary Materials Figures S1–S5), with the latter used for validation. 

### 3.2. Multiplex PCR

#### 3.2.1. Screening of Local Cultivated Varieties

**Figure 2:**
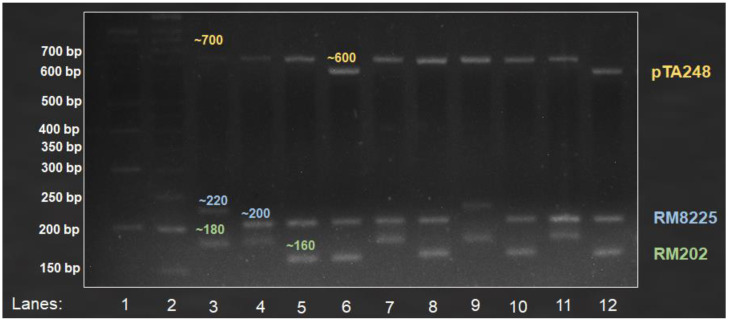
Amplified multiplex products from pTA248, RM8225, and RM202, associated with bacterial leaf blight (~600 bp (susceptible) and ~700 bp (resistance)), blast (~220 bp (susceptible) and ~200 bp (resistance)), and sheath blight (~180 bp (susceptible) and ~160 bp (resistance)) resistance genes, respectively. Lanes 1–2: 100 bp and 50 bp ladders; Lanes 3–12: MR219, Mahsuri Mutant, Pulut Hitam 9, Ria, MR167, MR185, MR220, MR106, MRQ74, and Pulut Malaysia 1.

**Figure 3:**
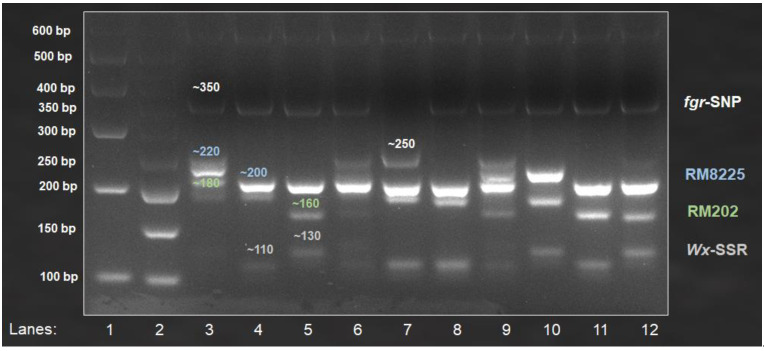
Amplified multiplex products from *fgr*-SNP, *Wx*-SSR, RM8225, and RM202, associated with fragrance (~250 bp (fragrant) and ~350 bp (nonfragrant)), amylose content (~130 bp (<25% amylose) and ~110 bp (>25% amylose)), blast (~220 bp (susceptible) and ~200 bp (resistance)), and sheath blight (~180 bp (susceptible) and ~160 bp (resistance)) resistance genes, respectively. Lanes 1–2: 100 bp and 50 bp ladders; Lanes 3–12: MR219, Mahsuri Mutant, Pulut Hitam 9, Ria, MRQ74, MR167, MR185, MR220, MR106, and Pulut Malaysia 1.

All the primers that were tested using uniplex PCR were then used to develop multiplex PCR assays. Table 5 shows the sizes of the amplified products of cultivated rice varieties from six multiplex PCRs (Supplementary Material Table S1). Gel images for two (Supplementary Materials Figures S6–S9) or more primer pairs showed clear resolution of products from all developed multiplex assays in 4% high-resolution agarose gel. This study developed two multiple assays with more than two primer sets; one of the assays used three sets of primers (pTA248, RM8225, and RM202) to represent each of the three biotic stresses (Figure 2), while the other used four sets of primers (*fgr*-SNP, *Wx*-SSR, RM8225, and RM202) to detect both quality traits and two biotic stresses (Figure 3).

#### 3.2.2. Screening of Local Weedy Rice Biotypes

**Figure 4:**
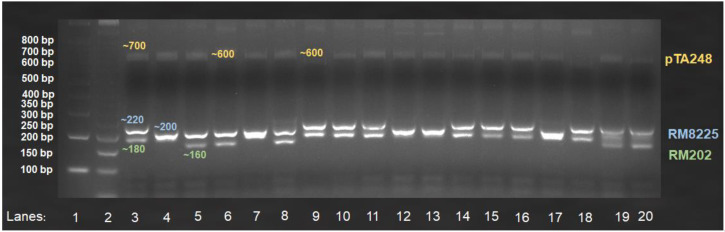
Examples of amplified multiplex pTA248, RM8225, and RM202 products separated using 4% high-resolution agarose gel electrophoresis at 120 V for 3 h, associated with bacterial leaf blight (~600 bp (susceptible) and ~700 bp (resistance)), blast (~220 bp (susceptible) and ~200 bp (resistance)), and sheath blight (~180 bp (susceptible) and ~160 bp (resistance)) resistance genes, respectively. Lanes 1–2: 100 bp and 50 bp ladders; Lanes 3–6 (rice checks): MR219, Mahsuri Mutant, Pulut Hitam 9, and Ria; Lanes 7–20 (weedy rice biotypes): WR20, WR21, WR22, WR23, WR24, WR25, WR26, WR27, WR28, WR29, WR30, WR31, WR07, and WR16.

A total of 100 weedy rice biotypes were tested for disease resistance traits using one of the developed multiplex assays, which included primer sets for blast (RM8225), sheath blight (RM202), and bacterial leaf blight (pTA248). The multiplex product sizes of the 100 weedy rice biotypes separated on 4% high-resolution agarose gels are provided in Supplementary Table S2. Figure 4 shows one of the gel images produced by the multiplex assay involving four local checks and fourteen weedy rice biotypes.

**Figure 5:**
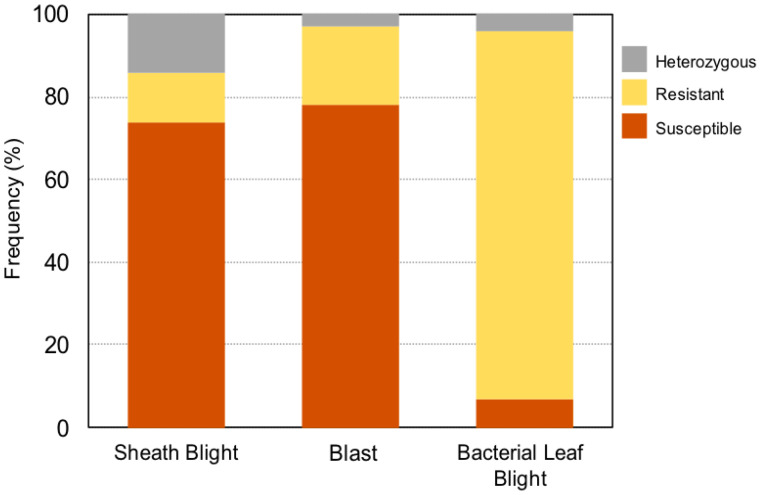
Frequency distribution of weedy rice biotypes associated with major rice diseases in Malaysia. Resistant and susceptible genotypes were determined based on molecular screening.

The genotyping analysis revealed that 12%, 19%, and 89% of the weedy rice biotypes were resistant to sheath blight, blast, and bacterial leaf blight, respectively (Figure 5). Sheath blight had the highest heterozygosity (14%), followed by bacterial leaf blight and blast.

**Figure 6:**
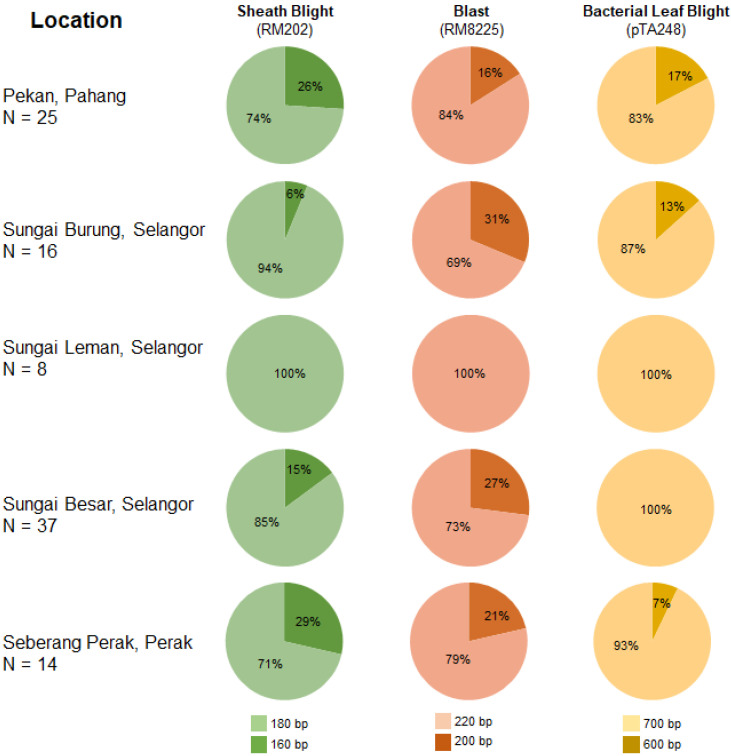
Allelic frequency of Malaysian weedy rice samples for markers associated with major biotic stresses.

Figure 6 shows the allelic frequency of markers RM202, RM8225, and pTA248 for the 100 weedy rice biotypes collected in various locations. All biotypes collected in Sungai Besar and Sungai Leman, Selangor, had 700 bp product sizes, indicating resistance to bacterial leaf blight. Among the studied weedy rice biotypes, the highest Shannon’s information index was 0.62 for blast disease detected by marker RM8225 in Sungai Burung, Selangor (Table 6).

**Figure 7:**
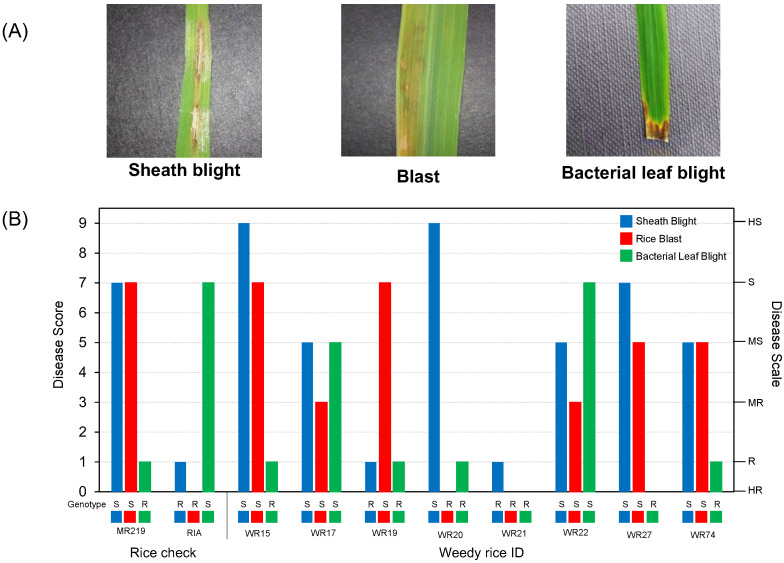
(**A**) Symptoms of sheath blight, blast, and bacterial leaf blight in weedy rice biotypes and (**B**) disease score, scale index, and genotype (S, susceptible; R, resistant) on two local rice checks, MR219 and Ria, and eight selected weedy rice samples.

Greenhouse phenotyping was performed to validate the genotyping results, primarily using the SES scale developed by IRRI for all three biotic stresses (sheath blight, blast, and bacterial leaf blight) [28]. Figure 7 shows the symptoms and genotype–phenotype analysis for our weedy rice population based on a random subset of eight weedy rice biotypes. The disease score, scale index, and genotypes of the selected biotypes were compared to two local rice checks, MR219 and Ria (Table 3, Figure 7B). The decision to use a subset to represent the weedy population was made following previous rice research, such as Tian et al. [36] and Li et al. [37], which employed 15 and 30 inoculated greenhouse-grown plants, respectively, to represent larger populations in their studies, which consisted of more than 400 accessions, for disease verification. Based on the reported sample group of the weedy population (Figure 7B), we obtained 75% accuracy in predicting the phenotypes of weedy rice biotypes using the genotype data from our developed multiplex assay. It is important to note that the percentage of accuracy can vary depending on a variety of factors, including the phenotyping scale [38]. According to Furbank and Tester [39], extensive phenotyping on a large scale is considered onerous. The cost of large-scale phenotyping may not be justified by the benefits it may provide [40].

## 4. Discussion

Rice breeders frequently aim to improve multiple key agronomic traits, including quality and disease resistance, via marker-assisted breeding, which typically involves the screening of a large number of breeding materials [19,41,42,43]. While many advanced methods for analyzing rice genotypes have been developed, such as capillary electrophoresis and direct DNA sequencing after PCR amplification, they are often expensive and require specialized tools or equipment (such as fluorescence readers and sequencers) that are not readily available in routine molecular laboratories, particularly in developing or underdeveloped countries [19]. In order to save the cost of genotyping, with no expense required to purify the DNA, we developed six convenient agarose-based multiple PCR assays to analyze multiple genes in a single PCR tube, which can determine up to four rice genes (Figure 2 and Figure 3) for major grain quality (amylose content and fragrance) and biotic stress resistance traits (bacterial leaf blight, blast, and sheath blight) where the alleles differ by less than 20 bp (Table 5). These multiplex assays can be used in a variety of rice breeding programs aimed at improving major grain quality, biotic stress resistance, or both. To test the effectiveness of the selected primer sets, uniplex PCRs were performed on local traditional and improved rice varieties with diverse grain quality and disease resistance backgrounds, and the results for the control varieties were validated through sequencing (Supplementary Materials Figures S1–S5). The uniplex PCR results were consistent with previous studies [19,20,41,44].

According to Vieira et al. [45], DNA-based markers such as simple sequence repeats (SSRs) and single-nucleotide polymorphisms (SNPs) are among the most dependable and stable tools for the discrimination of rice varieties. We discovered that the thermal cycling profile designed by McCouch et al. [25] to map rice SSRs works effectively for all of the multiplex assays developed in this study, which used several different molecular markers, including SSR, SNP, and sequence-tagged site (STS). The current study used a single PCR cycle and a fixed agarose gel preparation and running protocol, providing a universal genotyping assay for the target traits. This implies that the same protocol can be used to screen for other rice genes containing functional molecular markers. Regardless of the types of molecular markers used, the melting temperature (T_m_) for all six multiplex assays was 55 °C. Researchers can avoid testing an exhaustive set of primers for the reported traits by using the developed assays, which can be easily employed in current and future breeding programs in Malaysia and Asia. According to our genotyping results, traditional Malaysian rice has shown good resistance to the major diseases, particularly Pulut Hitam 9, which is resistant to all three diseases (Table 5; Figure 2 and Figure 3). Because Pulut Hitam 9 is a waxy rice, it can be used as a donor parent for the development of low-amylose or waxy rice with disease resistance [3,4,9].

Weedy rice (*O. sativa*), also known locally as “padi angin” (due to its easily shattered seeds), is one of the most dominant and competitive weed species found in rice planting areas worldwide [46]. Recent reports showed that weedy rice contains novel sources of disease resistance [14], including broad-spectrum resistance to blast, the *most* explosive and potentially damaging rice disease worldwide [47]. Our research revealed that the vast majority of the studied weedy rice biotypes were genetically resistant to bacterial leaf blight disease (Figure 5; Table S2). This indicates that weedy rice can be an effective option to screen the specific beneficial genes and cross-over with a rice cultivar to produce a new bacterial leaf blight resistant rice variety. To date, there are more than 42 resistance genes that have been identified and used in rice plant breeding from various *Oryza* spp. [48]. Among the bacterial leaf blight resistance genes identified is *Xa21*, which was originally introgressed from a wild rice accession, *O. longistaminata*, and mapped to chromosome 11 [49,50]. Additionally, the weedy rice biotypes in our study also demonstrated some level of resistance to blast and sheath blight (Figure 5). A recent study conducted by Goad et al. [51] suggested that blast and sheath blight resistance varies by genotype in weedy rice populations, with *indica* types being more resistant than *japonica* types. The greater the genetic diversity in a population, the higher the Shannon’s index [52]. In Sungai Leman, Selangor, Shannon’s information index was zero for all three diseases (Table 6). This could be because only eight samples (N = 8) were collected from this particular location, and the limited sample size might not accurately reflect the genetic diversity of the population [53]. We recommend that weedy population genetics studies use larger sample sizes to better capture the genetic diversity of populations in a specific location.

To the best of our knowledge, none of the six multiplex assays developed in this study were previously reported, particularly for screening weedy rice biotypes. Our universal multiplex protocol is rapid (detecting up to four genes in a single PCR run), easy to employ, and cost-effective because no specialized laboratory equipment is required. Our results are consistent with the findings of Duan et al. [54], who reported a high-specificity universal multiplex PCR assay capable of detecting multiple pathogens associated with human bacterial meningitis. We believe that agarose-gel-based multiplex PCR is one of the best approaches for genotyping a large number of parents (varieties), progeny, and/or weedy rice populations in molecular laboratories with limited resources. However, if funding is not a constraint, advanced genotyping methods such as capillary electrophoresis, real-time detection, or direct sequencing would be better alternatives, as they allow quick screening for alleles that differ by as little as 1 bp. The advanced methods are also slightly less labor-intensive because they can be automated and do not involve the use of agarose gel, although they require qualified laboratory personnel or skilled researchers to run the equipment and interpret the data [55,56]. Our study found that 89% of the tested weedy biotypes were resistant to bacterial leaf blight, indicating the potential to introduce resistance alleles from local weedy biotypes into cultivated rice varieties that are susceptible to the disease. Given that rice and weedy rice are closely related and have a low breeding barrier [53], we urge that the potential and genetic diversity of weedy rice be explored further for use in rice breeding programs.

## 5. Conclusions

In the postgenomic era, where functional markers are more widely available to breeders to employ, a simple and cost-effective genotyping method is in high demand, particularly for those working in developing and underdeveloped countries that have limited resources. Our agarose-based multiplex PCR assays require three pieces of conventional equipment: a PCR thermocycler, an electrophoresis system, and a gel imager, all of which are typically readily available in any molecular or genotyping laboratory. The assays have been shown to be effective for the molecular analysis of major grain quality and biotic stresses in both cultivated and weedy rice, regardless of the types of markers used. We believe that the developed assays will aid in accelerated marker-assisted breeding in many breeding programs throughout Asia. 

## Figures and Tables

**Table 1:** Details of molecular markers employed for PCR analysis.

Category	Trait	Locus/ Loci	Chr.	Marker Name	Ref	Primer Sequences (5’-3’)
Grain quality	Amylose levels	*Wx*	6	*Wx*-SSR	[20,21]	F’ -CTTTGTCTATCTCAAGACAC R’ -TTGCAGATGTTCTTCCTGATG
	Fragrance	*Fgr*	8	*fgr*-SNP	[20,21]	EAP -AGTGCTTTACAAAGTCCCGC ESP -TTGTTTGGAGCTTGCTGATG IFAP -CATAGGAGCAGCTGAAATA TATACC INSP -CTGGTAAAAAGATTATGGCTTCA
Biotic stresses	Bacterial leaf blight	*Xa21*	11	pTA248	[22]	F’ -AGACGCGGAAGGGTGGTTCCCGGA R’ -AGACGCGGTAATCGAAAGATGAAA
	Blast	*Piz*	6	RM8225	[23]	F’ -ATGCGTGTTCAGAAATTAGG R’ -TTGTTGTATACCTCATCGACAG
	Sheath blight	qSBR11-3, QRlh11	11	RM202	[24]	F’ -CAGATTGGAGATGAAGTCCTCC R’ -CCAGCAAGCATGTCAATGTA

Chr.—Chromosome; F’—Forward; R’—Reverse; EAP—External Antisense Primer; ESP—External Sense Primer; IFAP—Internal Fragrant Antisense Primer; INSP—Internal Nonfragrant Sense Primer; Ref—Reference(s).

**Table 2:** Description of thermal cycling parameters for uniplex and multiplex PCR reactions.

Optimization	Uniplex (Quality Traits)	Uniplex (Biotic Stress Traits)	Multiplex
Thermal cycling profile	Cheng et al. [20]	Mohd Hanafiah et al. [19]	McCouch et al. [25]
Initial denaturation	95 °C (4.00 min)	94 °C (5.00 min)	94 °C (5.00 min)
Cycle number	34	35	35
Denaturation	94 °C (0.75 min)	94 °C (1.00 min)	94 °C (1.00 min)
Annealing	55 °C (0.75 min)	55 °C (1.00 min)	55 °C (1.00 min)
Extension	72 °C (0.75 min)	72 °C (2.00 min)	72 °C (2.00 min)
Final extension	72 °C (5.00 min)	72 °C (5.00 min)	72 °C (5.00 min)
Concentration of each primer	0.4 µM (*Wx*-SSR or *fgr*-SNP)	0.4 µM (pTA248, RM8225, or RM202)	0.4 µM
Concentration of DNA template	50 ng	50 ng	50 ng
Final reaction volume	20 µL	20 µL	20 µL

**Table 3:** Local rice checks for selected quality and biotic stress traits and the expected sizes of their PCR products.

Trait	Primer	Positive Control	Description	Expected Product Size (bp)	Negative Control	Description	Expected Product Size (bp)
Amylose levels	*Wx*-SSR	MRQ74	>25% amylose	~110	MR219	<25% amylose	~130
Fragrance	*fgr*-SNP	MRQ74	Fragrant	~250	MR219	Nonfragrant	~350
Bacterial leaf blight	pTA248	MR219	Resistant	~700	Ria	Susceptible	~600
Blast	RM8225	Mahsuri Mutant; Ria	Resistant	~200	MR219	Susceptible	~220
Sheath blight	RM202	Pulut Hitam 9; Ria	Resistant	~160	MR219	Susceptible	~180

**Table 4:** Description of phenotype scoring for rice diseases according to the Standard Evaluation System (SES) for rice [28].

Biotic Stress	Score					
	0	1	3	5	7	9
Disease scale	Highly resistant (HR)	Resistant (R)	Moderately resistant (MR)	Moderately susceptible (MS)	Susceptible (S)	Highly susceptible (HS)
Blast	No lesion	Uniform or scattered brown specks	Small lesion ~1 mm in diameter	1–2 mm elliptical lesions	Broad spindle-shaped lesion with yellow, brown, or purple margin	Rapidly coalescing small, whitish, grayish, or bluish lesions without distinct margins
Sheath blight	No lesion	<20% lesion	20–30% lesion	31–45% lesion	46–65% lesion	>65% lesion
Bacterial leaf blight	No lesion	1–5% lesion	6–12% lesion	13–25% lesion	26–50% lesion	>50% lesion

**Table 5:** Amplified product size (bp) from uniplex PCR analysis of ten selected local rice varieties.

Trait Variety	Amylose Levels	Fragrance	Bacterial Leaf Blight	Blast	Sheath Blight
	*Wx*-SSR	*fgr*-SNP	pTA248	RM8225	RM202
MR219	~130 (NH)	~350 (NF)	~700 (R)	~220 (S)	~180 (S)
MRQ74	~110 (H)	~250 (F)	~700 (R)	~200 (R)	~180 (S)
Ria	~110 (H)	~350 (NF)	~600 (S)	~200 (R)	~160 (R)
Mahsuri Mutant	~110 (H)	~350 (NF)	~700 (R)	~200 (R)	~180 (S)
Pulut Hitam 9	~130 (NH)	~350 (NF)	~700 (R)	~200 (R)	~160 (R)
Pulut Malaysia 1	~130 (NH)	~350 (NF)	~600 (S)	~200 (R)	~160 (R)
MR106	~110 (H)	~350 (NF)	~700 (R)	~200 (R)	~160 (R)
MR167	~110 (H)	~350 (NF)	~700 (R)	~200 (R)	~180 (S)
MR185	~110 (H)	~350 (NF)	~700 (R)	~200 (R)	~160 (R)
MR220	~130 (NH)	~350 (NF)	~700 (R)	~220 (S)	~180 (S)

H: High amylose; NH: Non-high amylose; F: Fragrant; NF: Nonfragrant; R: Resistant; S: Susceptible.

**Table 6:** Genetic diversity of weedy rice samples based on three loci associated with sheath blight, blast, and bacterial leaf blight diseases in five different rice fields in Malaysia.

Location	Diseases	N	Na	Ne	I	Ho	He	uHe
Pekan, Pahang	Sheath blight	25	2.000	1.625	0.573	0.120	0.385	0.393
	Blast		2.000	1.368	0.440	0.080	0.269	0.274
	Bacterial leaf blight		2.000	1.403	0.462	0.000	0.287	0.294
Sungai Burung, Selangor	Sheath blight	16	2.000	1.133	0.234	0.000	0.117	0.121
	Blast		2.000	1.753	0.621	0.000	0.430	0.444
	Bacterial leaf blight		2.000	1.301	0.393	0.000	0.231	0.239
Sungai Leman, Selangor	Sheath blight	8	1.000	1.000	0.000	0.000	0.000	0.000
	Blast		1.000	1.000	0.000	0.000	0.000	0.000
	Bacterial leaf blight		1.000	1.000	0.000	0.000	0.000	0.000
Sungai Besar, Selangor	Sheath blight	37	2.000	1.339	0.420	0.027	0.253	0.257
	Blast		2.000	1.651	0.584	0.000	0.394	0.400
	Bacterial leaf blight		1.000	1.000	0.000	0.000	0.000	0.000
Seberang Perak, Perak	Sheath blight	14	2.000	1.690	0.598	0.286	0.408	0.423
	Blast		2.000	1.508	0.520	0.000	0.337	0.349
	Bacterial leaf blight		2.000	1.153	0.257	0.000	0.133	0.138

N: Sample size; Na: Number of alleles; Ne: Number of effective alleles; I: Shannon’s information index; Ho: Observed heterozygosity; He: Expected heterozygosity; uHe: Unbiased expected heterozygosity.

## Data Availability

Not applicable.

## Supplementary Materials

The following supporting information can be downloaded at: https://www.mdpi.com/article/10.3390/life12101542/s1, Figures S1–S5: Partial sequences and amplified PCR products of primer sets; Figures S6–S9: Amplified multiplex products from two primer sets associated with major rice quality and/or disease resistance traits; Table S1: Description of six PCR-based multiplex assays developed in this study; Table S2: Description of multiplex PCR results for 100 weedy rice biotypes.
