# Supplementary material for: Novel PCR-Based Multiplex Assays for Detecting Major Quality and Biotic Stress in Commercial and Weedy Rice
Source: Life (Basel). 2022 Oct 4;12(10):1542. doi: 10.3390/life12101542 (PMC9604669; doi:10.3390/life12101542)
Supplement: Supplementary file 1 [file life-12-01542-s001.zip › life-1951911-supplementary.pdf]

SUPPLEMENTARY MATERIALS

Traits	Sequence
>25% amylose rice	CAAATAACTGCAGTCTCTCTCTCTCTCTCTCT--------------GCTTCACTTCTCTGCTTGTGT
<25% amylose rice	CAAATAACTGCAGTCTCTCTCTCTCTCTCTCTCTCTCTCTCTCTCTCTGCTTCACTTCTCTGCTTGTGT
	********************************** *********************

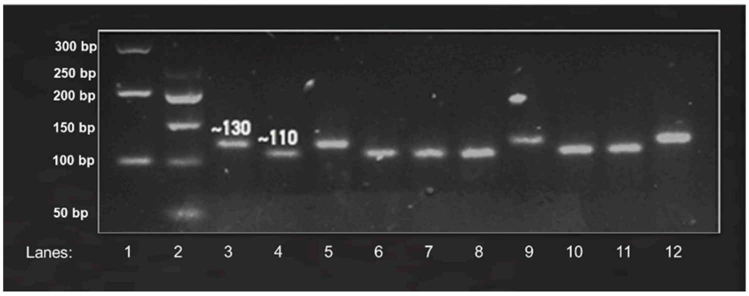

Figure S1. Partial sequences and amplified PCR products of *Wx*- SSR primers for amylose content. Lanes 1 - 2: 100 bp and 50 bp ladders; Lanes 3 - 12: MR219, Mahsuri Mutant, Pulut Hitam 9, Ria, MR167, MR185, MR220, MR106, MRQ74, and Pulut Malaysia 1

Traits	Sequence
Non-fragrant rice	AGGTTGCATTTACTGGGAGTTATGAAACTGGTAAAAAGATTATGGCTTCAGCTGCTCCTATGGTTAAG
Fragrant rice	AGGTTGCATTTACTGGGAGTTATGAAACTGGTATATA--------TTTCAGCTGCTCCTATGGTTAAG
	********************************* * * **********************

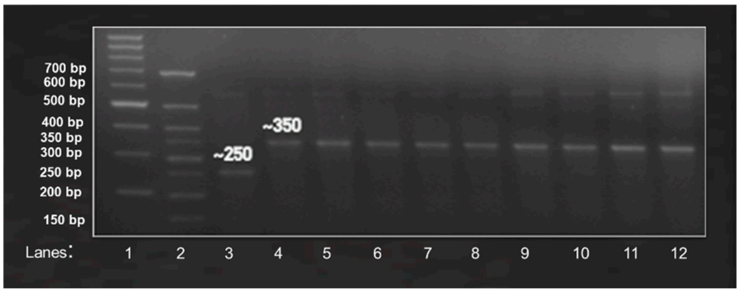

Figure S2. Partial sequences and amplified PCR products of *fgr*-SNP primers for fragrance. Lanes 1 - 2: 100 bp and 50 bp ladders; Lanes 3 - 12: MRQ74, MR219, Mahsuri Mutant, Pulut Hitam 9, Ria, MR167, MR185, MR220, MR106, and Pulut Malaysia 1

Traits	Sequence
BLB resistant	AGGCCACCTCAGGATAACAAAAAGTGGCAGACCTATTTCTTTTCCGCATATGGACCAAAAGGAAAAAA
BLB susceptible	AGGCCACCTCAGGATAACAAA---
	********************************** *********************
BLB resistant	GAGTAAACTCCTGCGAAAATGGCTAAGGCCACCTCAGGATAACAAACTGTGGCAG
BLB susceptible	--CTGTGGCAG
	*********

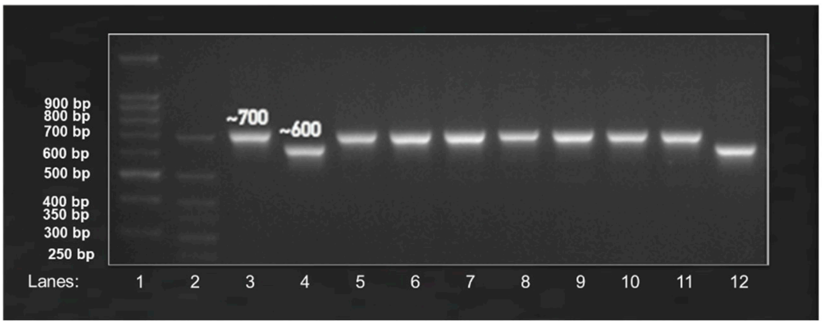

Figure S3. Partial sequences and amplified PCR products of pTA248 primers for bacterial leaf blight resistance. Lanes 1 - 2: 100 bp and 50 bp ladders; Lanes 3 - 12: MR219, Ria, Mahsuri Mutant, Pulut Hitam 9, MR167, MR185, MR220, MR106, MRQ74, and Pulut Malaysia 1

Traits	Sequence
Blast resistant	TCTTCTTCTTCTTCTTCTTCT------------------------TTCCATTTTCCCATTTTCTA
Blast susceptible	TCTTCTTCTTCTTCTTCTTCTTCTTCTTCTTCTTCTTCTTCTTCTTTCCATTTTCCCATTTTCTA
	********************* ********************

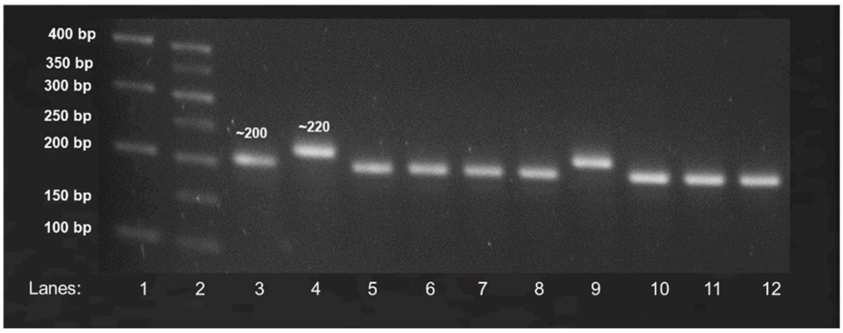

Figure S4. Amplified PCR products of RM8225 primers for blast resistance. Lanes 1 - 2: 100 bp and 50 bp ladders; Lanes 3 - 12: Mahsuri Mutant, MR219, Pulut Hitam 9, Ria, MR167, MR185, MR220, MR106, MRQ74, and Pulut Malaysia 1

Traits	Sequence
SB resistant	ACTCATCCTCTCTCTCTCTCTCTCTCTCTCTCTCTCTCT----------------------TCTAACATA
SB susceptible	ACTCATCCTCTCTCTCTCTCTCTCTCTCTCTCTCTCTCTCTCTCTCTCTCTCTCTCTCTTCTAACATA
	*************************************** *********

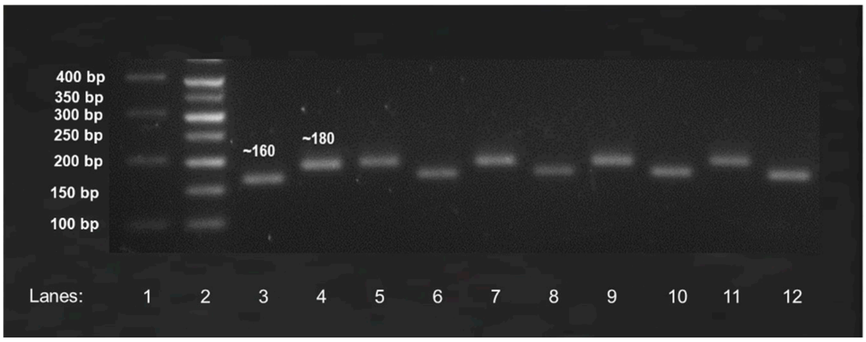

Figure S5. Partial sequences and amplified PCR products of RM202 primers for sheath blight resistance. Lanes 1 - 2: 100 bp and 50 bp ladders; Lanes 3 - 12: 1: Pulut Hitam 9, MR219, Mahsuri Mutant, Ria, MR167, MR185, MR220, MR106, MRQ74, and Pulut Malaysia 1

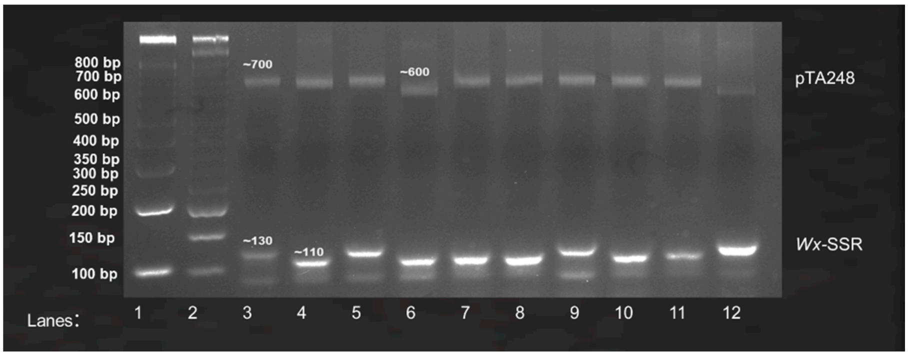

Figure S6. Amplified multiplex products from *Wx*-SSR and pTA248 associated with amylose content [~110 bp (>25% amylose); ~130 bp (<25% amylose)] and bacterial leaf blight resistance [~600 bp (susceptible); ~700 bp (resistant)] genes, respectively. Lanes 1 - 2: 100 bp and 50 bp ladders; Lanes 3 - 12: MR219, Mahsuri Mutant, Pulut Hitam 9, Ria, MR167, MR185, MR220, MR106, MRQ74, and Pulut Malaysia 1

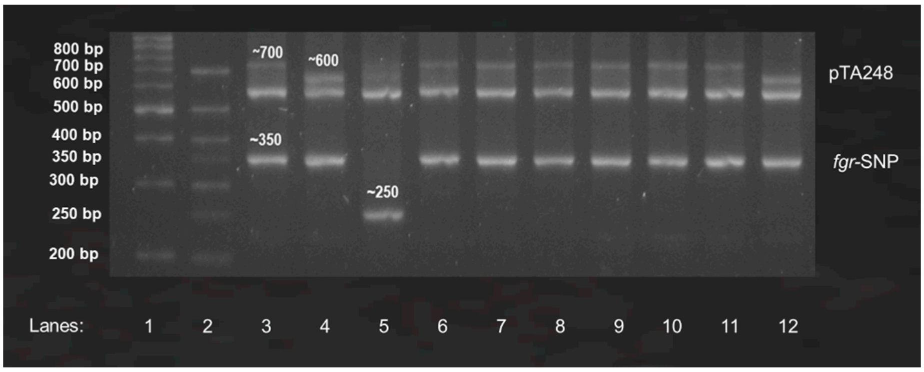

Figure S7. Amplified multiplex products from *fgr*-SNP and pTA248 associated with fragrance [~250 bp (fragrant); ~350 bp (non-fragrant)] and bacterial leaf blight resistance [~600 bp (susceptible); ~700 bp (resistant)] genes, respectively. Lanes 1 - 2: 100 bp and 50 bp ladders; Lanes 3 - 12: MR219, Ria, MRQ74, Mahsuri Mutant, Pulut Hitam 9, MR167, MR185, MR220, MR106, and Pulut Malaysia 1

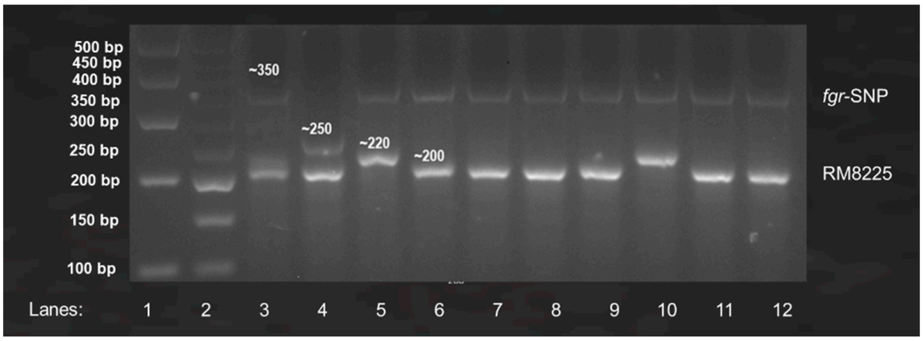

Figure S8. Amplified multiplex products from *fgr*-SNP and RM8225 associated with fragrance [~250 bp (fragrant); ~350 bp (non-fragrant)] and blast resistance [~200 bp (resistant); ~220 bp (susceptible)] genes, respectively. Lanes 1 - 2: 100 bp and 50 bp ladders; Lanes 3 - 12: Mahsuri Mutant, MRQ74, MR219, Pulut Hitam 9, Ria, MR167, MR185, MR220, MR106, and Pulut Malaysia 1

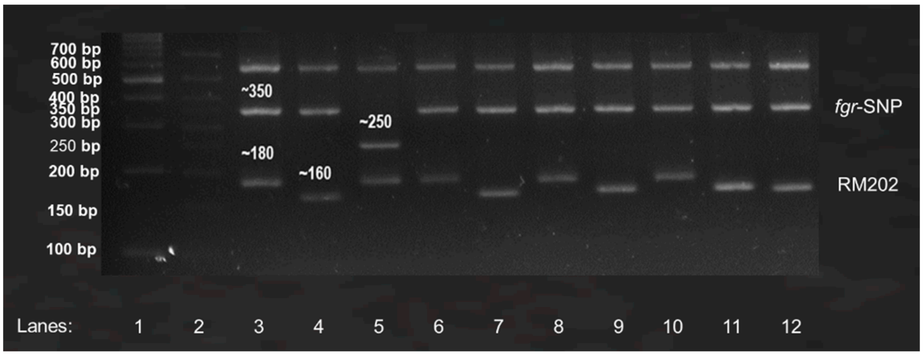

Figure S9. Amplified multiplex products from *fgr*-SNP and RM202 associated with fragrance [~250 bp (fragrant); ~350 bp (non-fragrant)] and sheath blight resistance [~160 bp (resistant); ~180 bp (susceptible)] genes, respectively. Lanes 1 - 2: 100 bp and 50 bp ladders; Lanes 3 - 12: MR219, Pulut Hitam 9, MRQ74, Mahsuri Mutant, Ria, MR167, MR185, MR220, MR106, and Pulut Malaysia 1

Table S1: Description of six PCR-based multiplex assays developed in this study

Variety	Two primer sets				Three primer sets	Four primer sets
	Wx-SSR + pTA248	*fgr*-SNP + pTA248	*fgr*-SNP + RM8225	*fgr*-SNP + RM202	RM202 +RM8225 +pTA248	*Wx*-SSR +RM202 +RM8225 +*fgr*-SNP
MR219	~130; ~700	~350; ~700	~350; ~220	~350; ~180	~180; ~220; ~700	~130; ~180; ~220; ~350
MRQ74	~110; ~700	~250; ~700	~250; ~200	~250; ~180	~180; ~200; ~700	~110; ~180; ~200; ~25 0
Ria	~110; ~600	~350; ~600	~350; ~200	~350; ~160	~160; ~200; ~600	~110; ~160; ~200; ~350
Mahsuri Mutant	~110; ~700	~350; ~700	~350; ~200	~350; ~180	~180; ~200; ~700	~110; ~180; ~200; ~350
Pulut Hitam 9	~130; ~700	~350; ~700	~350; ~200	~350; ~160	~160; ~200; ~700	~130; ~160; ~200; ~350
Pulut Malaysia 1	~130; ~600	~350; ~600	~350; ~200	~350; ~160	~160; ~200; ~600	~130; ~160; ~200; ~350
MR106	~110; ~700	~350; ~700	~350; ~200	~350; ~160	~160; ~200; ~700	~110; ~160; ~200; ~350
MR167	~110; ~700	~350; ~700	~350; ~200	~350; ~180	~180; ~200; ~700	~110; ~180; ~200; ~350
MR185	~110; ~700	~350; ~700	~350; ~200	~350; ~160	~160; ~200; ~700	~110; ~160; ~200; ~350
MR220	~130; ~700	~350; ~700	~350; ~220	~350; ~180	~180; ~220; ~700	~130; ~180; ~220; ~350

Table S2. Description of multiplex PCR results for 100 weedy rice biotypes

Biotypes	Multiplex product size	Multiplex description		
	RM202+RM8225+pTA248	Sheath blight	Blast	Bacterial leaf blight
**WR01**	~180;~220;~700	S	S	R
**WR02**	~160, ~180;~220;~700	H	S	R
**WR03**	~180;~220;~700	S	S	R
**WR04**	~160,~180;~220;~600	H	S	S
**WR05**	~180;~220;~600	S	S	S
**WR06**	~160;~220,~700	R	S	R
**WR07**	~160, ~180;~220; ~700	H	S	R
**WR08**	~160;~220;~700	S	S	R
**WR09**	~180;~220;~700	S	S	R
**WR10**	~180;~220;~700	S	S	R
**WR11**	~180;~220;~700	S	S	R
**WR12**	~180;~220;~700	S	S	R
**WR13**	~180;~220;~700	S	S	R
**WR14**	~180;~220	S	S	NA
**WR15**	~180;~220;~700	S	S	R
**WR16**	~160,~180;~220;~700	H	S	R
**WR17**	~180;~220;~600	S	S	S
**WR18**	~180;~200;~220;~700	S	H	R
**WR19**	~160;~220;~700	R	S	R
**WR20**	~180;~200;~700	S	R	R
**WR21**	~160;~200;~700	R	R	R
**WR22**	~180;~220;~600	S	S	S
**WR23**	~180;~220;~700	S	S	R
**WR24**	~180;~220;~700	S	S	R
**WR25**	~180;~200;~700	S	R	R
**WR26**	~180;~200;~700	S	R	R
**WR27**	~180;~220;~700	S	S	R
**WR28**	~180;~220;~700	S	S	R
**WR29**	~180;~220;~700	S	S	R
**WR30**	~180;200;700	S	R	R
**WR31**	180;220;600	S	S	S
**WR32**	180;200;700	S	R	R
**WR33**	~180;~200;~700	S	R	R
**WR34**	~180;~220;~700	S	S	R
**WR35**	~160,~180;~220;~700	H	S	R
**WR36**	~160;~200;~700	R	S	R
**WR37**	~180;~200;~700	S	R	R
**WR38**	~180;~220;~700	S	S	R
**WR39**	~180;~220;~700	S	S	R
**WR40**	~180;~220;~700	S	S	R
**WR41**	~180;~220;~600	S	S	S
**WR42**	~180;~220;~700	S	S	R
**WR43**	~180;~220;~700	S	S	R
**WR44**	~180;~220;~700	S	S	R
**WR45**	~180;~220;~700	S	S	R
**WR46**	180;220;700	S	S	R

**WR47**	~180;~220;~700	S	S	R
**WR48**	~160,~180;~220;~700	H	S	R
**WR49**	~160,~180;~220;~700	H	S	R
**WR50**	~180;~220;~700	S	S	R
**WR51**	~180;~220;~700	S	S	R
**WR52**	~180;~220;~700	S	S	R
**WR53**	~180;~200;~700	S	R	R
**WR54**	~180;~220;~700	S	S	R
**WR55**	~180;~220;~700	S	S	R
**WR56**	~180;~200;~700	S	R	R
**WR57**	~180;~220;~700	S	S	R
**WR58**	~180;~200;~700	S	R	R
**WR59**	~160,~180;~220;~700	H	S	R
**WR60**	~180;~200;~700	S	R	R
**WR61**	~180;~220;~700	S	S	R
**WR62**	~160;~220;~700	R	S	R
**WR63**	~180;~220;~700	S	S	R
**WR64**	~180;~220;~700	S	S	R
**WR65**	~180;~200;~700	S	R	R
**WR66**	~160,~180;~200;~700	H	R	R
**WR67**	~180;~220;~700	S	S	R
**WR68**	~180;~220;~700	S	S	R
**WR69**	~180;~220;~700	S	S	R
**WR70**	~180;~220;~700	S	S	R
**WR71**	~180;~200;~700	S	R	R
**WR72**	~180;~220;~700	S	S	R
**WR73**	~180;~200;~700	S	R	R
**WR74**	~180;~220;~700	S	S	R
**WR75**	~180;~220;~700	S	S	R
**WR76**	~160;~220;~700	R	S	R
**WR77**	~180;~220;~700	S	S	R
**WR78**	~180;~220;~700	S	S	R
**WR79**	~180;~220;~700	S	S	R
**WR80**	~180;~220;~700	S	S	R
**WR81**	~160;~220;~700	R	S	R
**WR82**	~180;~~220;~700	S	S	R
**WR83**	~180;~~220;~700	S	S	R
**WR84**	~160;~180;~220;~700	H	S	R
**WR85**	~160;~220;~700	R	S	R
**WR86**	~160;~200;~700	R	R	R
**WR87**	~160,~180;~220;700	H	S	R
**WR88**	~180;~220;~700	S	S	R
**WR89**	~160;~200;~700	R	R	R
**WR90**	~160,~180;~220;700	H	S	R
**WR91**	~160,~180;~220;700	H	S	R
**WR92**	~160;~200;~700	R	R	R
**WR93**	~180;~220;~700	S	S	R
**WR94**	~180;~220;~700	S	S	R
**WR95**	~180;~200,~220;~700	S	H	R
**WR96**	~180;~220;~700	S	S	R
**WR97**	~160;~220;~700	R	S	R
**WR98**	~180;~220;~600	S	S	S
**WR99**	~180;~220;~700	S	S	R
**WR100**	~180;~220;~700	S	S	R
